# Combining iterative metal artifact reduction and virtual monoenergetic images severely reduces hip prosthesis-associated artifacts in photon-counting detector CT

**DOI:** 10.1038/s41598-023-35989-6

**Published:** 2023-06-02

**Authors:** Yannik C. Layer, Narine Mesropyan, Patrick A. Kupczyk, Julian A. Luetkens, Alexander Isaak, Tatjana Dell, Ulrike I. Attenberger, Daniel Kuetting

**Affiliations:** Department of Diagnostic and Interventional Radiology, University Hospital Bonn, Venusberg-Campus 1, 53127 Bonn, Germany

**Keywords:** Computed tomography, Medical imaging

## Abstract

Aim of this study was to assess the impact of virtual monoenergetic images (VMI) in combination and comparison with iterative metal artifact reduction (IMAR) on hip prosthesis-associated artifacts in photon-counting detector CT (PCD-CT). Retrospectively, 33 scans with hip prosthesis-associated artifacts acquired during clinical routine on a PCD-CT between 08/2022 and 09/2022 were analyzed. VMI were reconstructed for 100–190 keV with and without IMAR, and compared to polychromatic images. Qualitatively, artifact extent and assessment of adjacent soft tissue were rated by two radiologists using 5-point Likert items. Quantitative assessment was performed measuring attenuation and standard deviation in most pronounced hypodense and hyperdense artifacts, artifact-impaired bone, muscle, vessels, bladder and artifact-free corresponding tissue. To quantify artifacts, an adjusted attenuation was calculated as the difference between artifact-impaired tissue and corresponding tissue without artifacts. Qualitative assessment improved for all investigated image reconstructions compared to polychromatic images (PI). VMI_100keV_ in combination with IMAR achieved best results (e.g. diagnostic quality of the bladder: median PI: 1.5 (range 1–4); VMI_100keV+IMAR_: 5 (3–5); p < 0.0001). In quantitative assessment VMI_100keV_ with IMAR provided best artifact reduction with an adjusted attenuation closest to 0 (e.g. bone: PI: 302.78; VMI_100keV+IMAR_: 51.18; p < 0.0001). The combination of VMI and IMAR significantly reduces hip prosthesis-associated artifacts in PCD-CT and improves the diagnostic quality of surrounding tissue.

## Introduction

Hip replacements, ranging among the most common orthopedic prostheses, typically lead to extensive artifacts in CT and therefore pose a common challenge in diagnostic imaging^1,2^. The severity of artifacts is influenced by image acquisition, image reconstruction parameters and composition of the metal implants^3^. Optimization of acquisition protocols and reconstruction parameters with use of high tube voltage, high tube current, narrow collimation and increased slice thickness allows for significant reduction of artifacts, however frequently does not suffice^3^. To enable diagnostic readability of artifact-impaired images further artifact reduction is needed.

Dual-energy CT (DECT) has been proven to reduce artifacts caused by arthroplasty^4^. Additionally, DECT-derived virtual monoenergetic images (VMI) have been shown to reduce artifact extent, however, lead to reduced contrast at higher keV^5,6^. Still, high keV VMI offer only limited artifact reduction in larger implants with the concurrent disadvantage of reduced contrast. Another possible adaption for reduction of metal artifacts are iterative metal artifact reduction algorithms (IMAR)^7,8^. Especially the combination of VMI and IMAR showed excellent results in previous DECT studies^9^. In 2021 the first PCD-CT was licensed for clinical use^10^. With direct conversion of every single photon into energy signals and the omittance of scintillators and detector septa, PCD-CT achieves higher spatial resolution, reduced radiation dose and less artifacts compared to energy-integrating detector CT (EID-CT)^11,12^. Especially high threshold images with tin filtration offer the prospect of substantial artifact reduction^13,14^.

Despite these promises, there are no clinical studies on the performance of VMI and IMAR in PCD-CT. Therefore, the aim of our study was to assess the impact of VMI and IMAR on image quality improvement in PCD-CT with hip prostheses associated artifacts.

## Materials and methods

The local institutional review board of the University Hospital Bonn approved this study (Ethics Committee of the Medical Faculty of the University of Bonn; reference number 426/22). The Ethics Committee of the Medical Faculty of the University of Bonn waived the need of informed consent. All scans were acquired during clinical routine and no scan was performed solely for research purposes. The study was conducted in accordance with the Declaration of Helsinki and its amendments.

Patients with hip implants and associated artifacts receiving abdominal/pelvic scan between 08/2022 and 09/2022 were included in this retrospective, single-center study. Scans were performed on a clinical PCD-CT (NAEOTOM Alpha, Siemens Healthcare GmbH, Erlangen, Germany). Inclusion criteria included the presence of hip prostheses and the acquisition of a spectral post-processing (SPP) enabled image data set with standard protocol as described below. Scans were performed for various indications.

### Imaging protocol

A weight-adapted volume of iodine based contrast agent (Accupaque 300 mg/ml, GE Healthcare Buchler GmbH & Co. KG, Braunschweig, Germany) was applied intravenously with a flow rate of 3 ml/s followed by a bolus of 40 ml of physiologic saline solution.

Scan parameters were a tube voltage of 120 kVp with activated automatic tube current modulation, a pitch of 0.8 and a gantry rotation time of 0.5 s. Detector collimation was 144 × 0.4 mm. Reconstruction parameters were 1 mm slice thickness with an increment of 0.7 mm. Scans were performed in a head-first supine position. A regular kernel (QR40; Siemens Healthcare GmbH, Erlangen, Germany) and Quantum Iterative Reconstruction (QIR Level 3; Siemens Healthcare GmbH, Erlangen, Germany) was used for image reconstruction.

VMI were reconstructed in axial view for 100–190 keV in an interval of 10 keV using dedicated software (syngo.via VB 60, Monoenergetic Plus; Siemens Healthcare GmbH, Erlangen, Germany). Iterative metal artifact reduction optimized for hip prosthesis (iMAR, Siemens Healthcare GmbH, Erlangen, Germany) is commercially available and was used as supplied by the vendor.

### Quantitative image analysis

Quantitative assessment of polychromatic and virtual monochromatic images was performed by region of interest (ROI) based attenuation analysis. ROIs were placed in the most pronounced hypodense and hyperdense artifacts using a conventional clinical DICOM viewer (Deep Unity R20 XX; Dedalus HealthCare GmbH, Bonn, Germany). Furthermore, values and standard deviation of X-ray attenuation was evaluated in muscle tissue, bone and bladder tissue with and without presence of artifacts. Hereby, differences in attenuation and gradient of attenuation of tissue were measured and an adjusted attenuation and image noise assessed as proposed previously^15^. Adjusted attenuation was assessed to differentiate artifact reduction from regular changes in Hounsfield units (HU) for differing VMI energy levels. Adjusted attenuation was calculated as the difference of tissue impaired by artifacts and without artifact impairment. Image noise is higher in images with presence of artifacts and VMI of high keV, therefore we calculated an adjusted image noise using the difference of standard deviation between muscle tissue in HU values in areas with and without artifacts. Adjusted attenuation of 0 indicates optimal artifact reduction, values above 0 indicate insufficient artifact reduction and values below 0 an overcorrection of the artifact correction. All measurements were performed for polychromatic and virtual monochromatic images from 100 to 190 keV in steps of 10 keV.

### Qualitative image analysis

Two radiologists with two (YCL) and eleven (DK) years of experience in abdominopelvic CT evaluated the CT images independently regarding artifact extent of hyperdense and hypodense artifacts as well as assessment of surrounding tissue using a five-point Likert grading scale. Rating of artifacts was defined as follows: (1) excessive artifacts; (2) pronounced artifacts; (3) moderate artifacts; (4) minor artifacts; and (5) artifacts are absent. For assessment of diagnostic quality of bone, muscle, vessels, bladder and soft tissue, the following Likert scale was used: (1) highly restricted diagnostic interpretability; (2) restricted diagnostic interpretability; (3) moderate diagnostic interpretability; (4) minor restrictions on diagnostic interpretability; and (5) unrestricted diagnostic interpretability. Additionally, both readers had to select the reconstruction with the best diagnostic quality for each patient. Polychromatic images as well as VMI with 100 keV, 130 keV, 160 keV and 190 keV were rated.

### Statistical analysis

All statistical analyses were conducted using IBM SPSS Version 27 (IBM Corp., Armonk, NY, USA). Graphs were carried out using the software GraphPad PRISM Version 6.02 (GraphPad Software, San Diego, CA, USA). Quantitative results are stated as mean and standard deviation. Wilcoxon signed-rank test was used for statistical analysis of quantitative image parameters. Qualitative results are expressed as median with interquartile range (IQR). Interrater reliability was assessed using the intraclass correlation coefficient (ICC). ICC estimates and their 95% confident intervals (CI) were calculated based on a mean-rating (k = 2), consistency, two-way mixed-effects model. p values below 0.05 were considered significant.

## Results

### Participant characteristics

**Figure 1:**
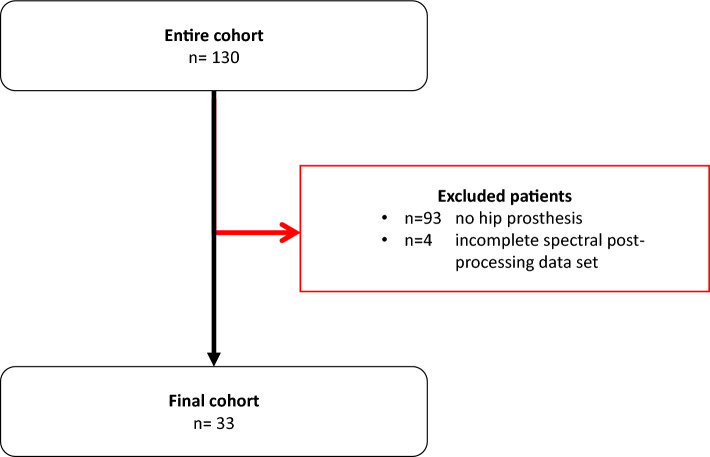
Patient flow chart. Between 08/2022 and 09/2022, a total of 130 patients received scans of the abdominal/pelvic region on a PCD-CT at our institute. 93 of these patients were excluded from analysis as they had no hip implants and 4 patients were excluded from analysis as they were missing a complete SPP image data set.

Between 08/2022 and 09/2022, a total of 130 patients received scans of the abdominal/pelvic region on a PCD-CT at our institute. 97 of these patients were excluded from analysis as they had no hip implants or were missing a complete SPP image data set (Fig. 1). Overall, 33 patients were included in the analysis (17 female, 51.52%) with an average age of 76 (range 61–91) years. Mean DLP was 515.58 mGy*cm and mean CTDI_vol_ was 7.78 mGy.

### Quantitative image analysis

**Figure 2:**
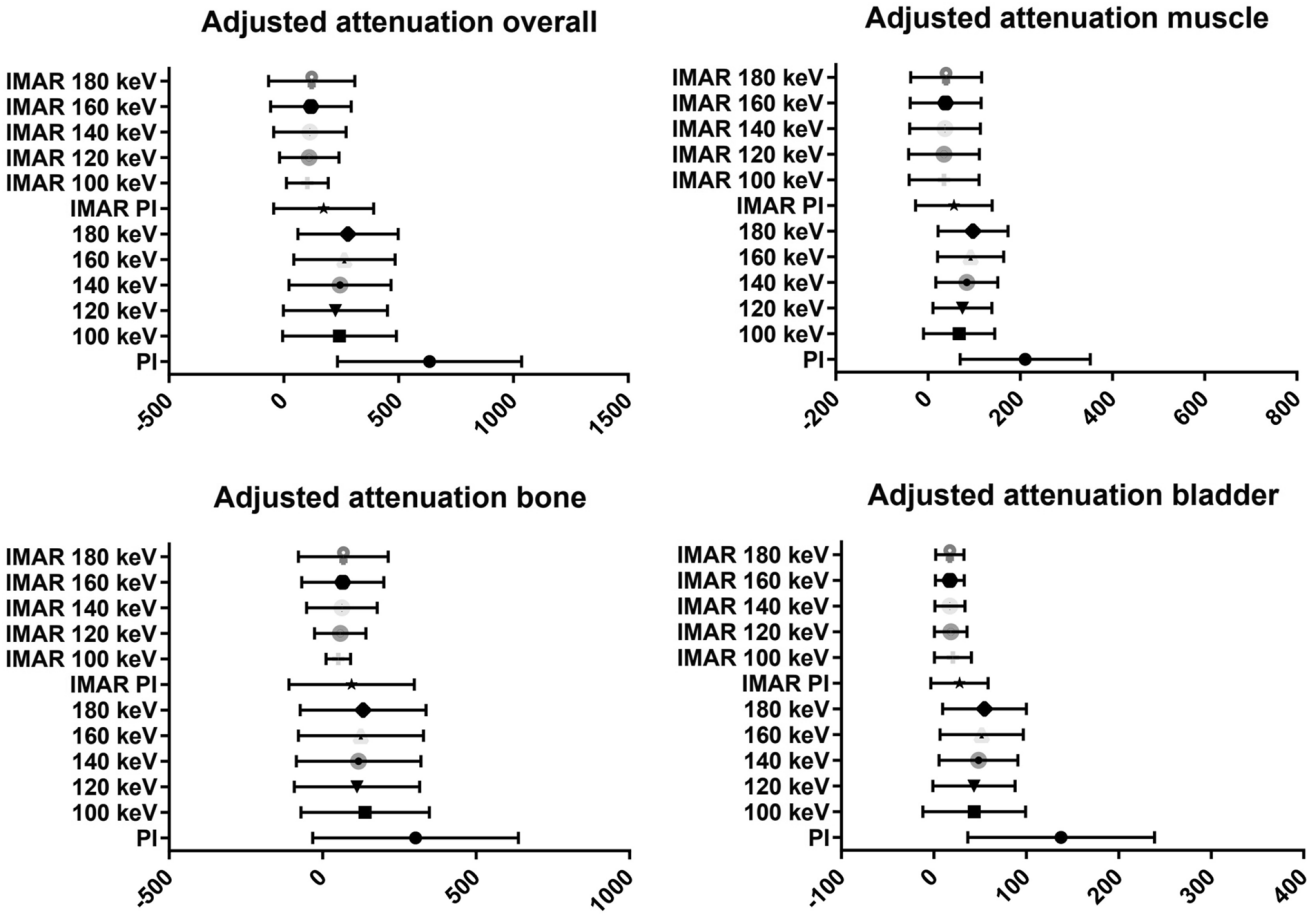
Mean adjusted attenuation and standard deviation for bladder, bone, muscle and overall analyzed tissue. Adjusted attenuation was calculated as difference between artifact-impaired tissue and corresponding tissue without artifacts. Values closest to zero show most favorable artifact reduction. VMI_100keV+IMAR_ showed the best adjusted attenuation overall compared to PI with the closest value to 0 (overall PI: 634.66; VMI_100keV+IMAR_: 102.81; p < 0.0001).

**Figure 3:**
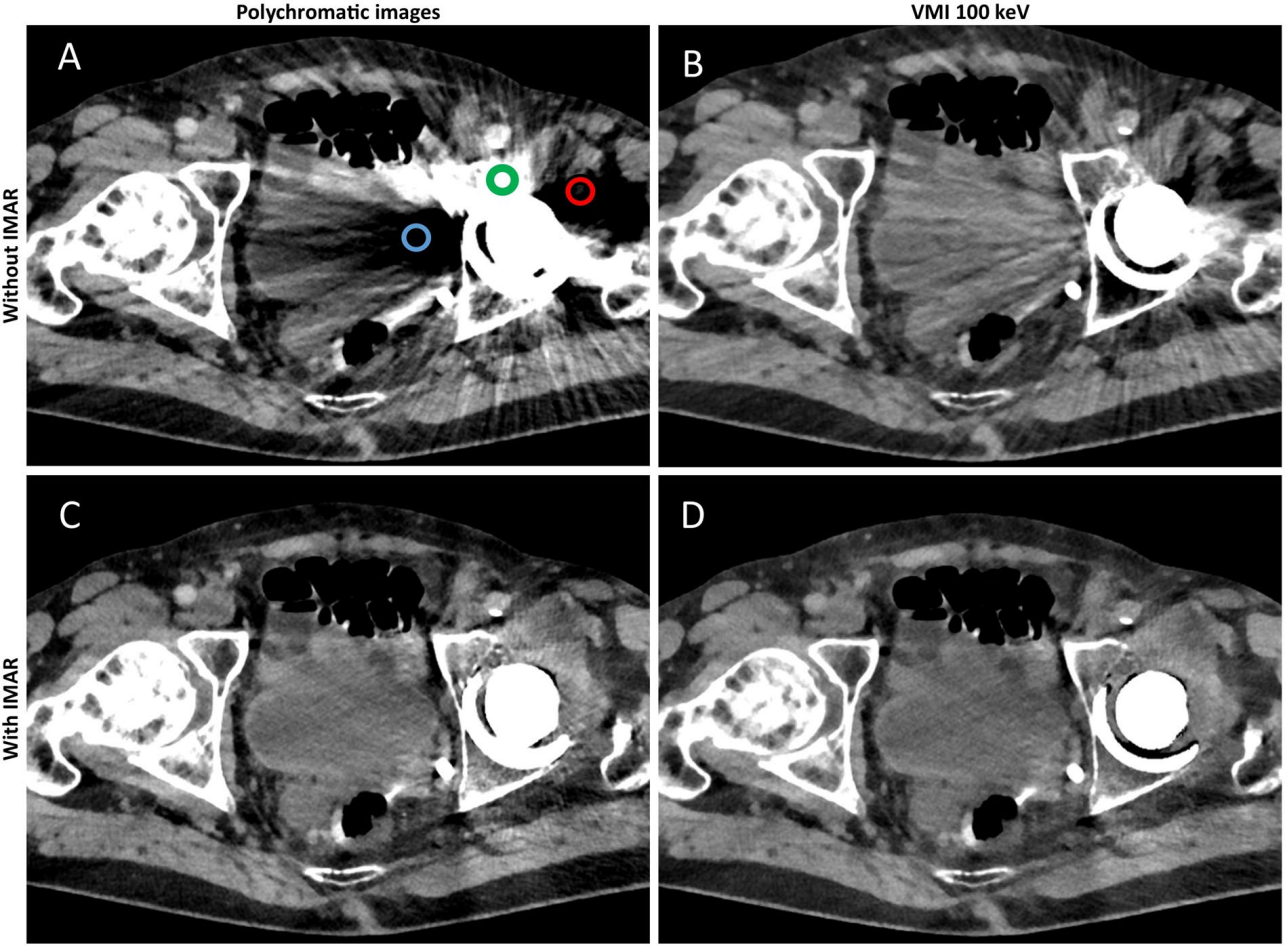
Representative axial polychromatic image (**A**), virtual monoenergetic image (VMI) of 100 keV (**B**), polychromatic image with iterative metal artifact reduction (IMAR) (**C**), and VMI of 100 keV in combination with IMAR (**D**) acquired with a photon-counting detector CT (PCD-CT) (window width/window level: 300/40 HU). Examples of ROI-Placement for artifacts in bone tissue (green), muscle tissue (red) and bladder tissue (blue) are shown in A. ROIs for corresponding artifact-free tissue are placed in slices in corresponding tissue without artifacts. Artifacts caused by hip prosthesis are most sufficiently reduced with the combination of VMI and IMAR.

**Figure 4:**
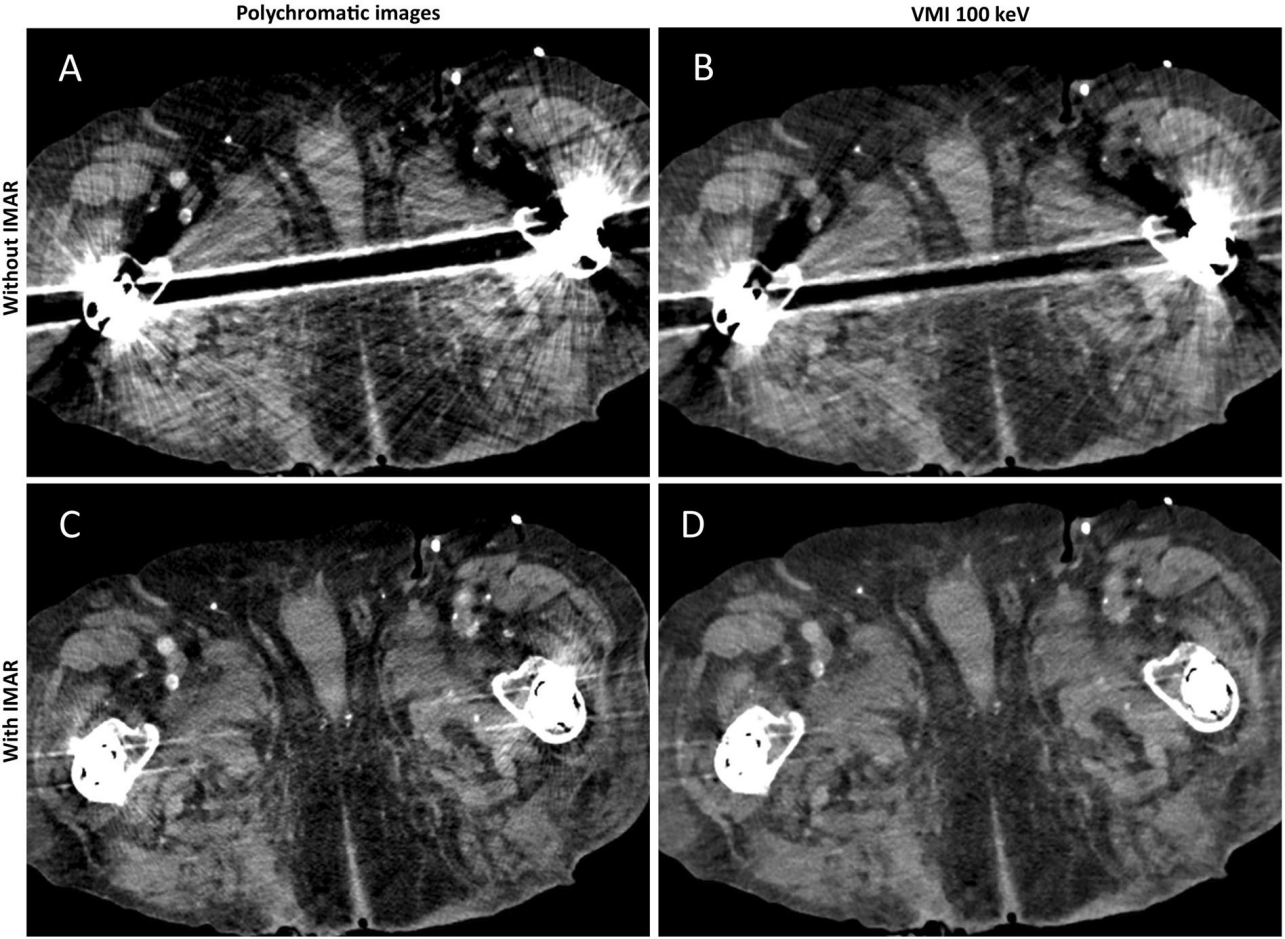
Representative axial polychromatic image (**A**), virtual monoenergetic image (VMI) of 100 keV (**B**), polychromatic image with iterative metal artifact reduction (IMAR) (**C**), and VMI of 100 keV in combination with IMAR (**D**) acquired with a photon-counting detector CT (PCD-CT) (window width/window level: 300/40 HU). Pronounced artifacts of bilateral hip prosthesis are sufficiently reduced with the combination of VMI and IMAR.

Results of quantitative analysis are summarized in Supplemental Information 1 and Fig. 2; Fig. 3 shows typical reconstructions of a pelvic CT with unilateral hip replacement and Fig. 4 shows representative images of a pelvic CT with bilateral hip replacement using VMI and IMAR. VMI_100keV+IMAR_ showed the best adjusted attenuation overall compared to PI with the closest value to 0 (Overall PI: 634.66; VMI_100keV+IMAR_: 102.81; p < 0.0001). Mean attenuation of hyperattenuating artifacts lowered in VMI compared to polychromatic images (PI: 661.58 ± 159.78; VMI_190keV_: 56.79 ± 96.82; p < 0.00001), in particular for the combination VMI and IMAR (VMI_190keV+IMAR_: 6.23 ± 69.91; p < 0.0001) (Supplemental Information 1).

Hypoattenuating artifacts showed an elevation of HU values in VMI (PI: − 424.97 ± 79.16; VMI_190keV_: − 71.63 ± 75.53; p = 0.0003), with increasingly higher values for higher keV reconstructions. The combination of VMI and IMAR showed most extensive artifact reduction with positive CT numbers for hypodense artifacts (VMI_100keV+IMAR_: 10.69 ± 27.12; VMI_190keV+IMAR_: 18.83 ± 26.35; p < 0.0001).

Adjusted image noise decreased for all reconstructions with the lowest value for VMI_110keV+IMAR_ (PI: 48.15; VMI_110keV+IMAR_: 8.37; p < 0.0001), as stated in Table 1. Mean ROI size was 50.34 mm^2^.

**Table 1:** Mean adjusted noise and adjusted attenuation values and standard deviation within defined regions of interest for polyenergetic reconstruction (PI) and virtual monoenergetic images (VMI) with and without iterative metal artifact reduction (IMAR).

	Adjusted noise	Adjusted attenuation (muscle)	Adjusted attenuation (bone)	Adjusted attenuation (bladder)	Adjusted attenuation (overall)
PI	50.40 ± 6.98	210.92 ± 24.68	302.78 ± 58.39	120.96 ± 18.87	634.66 ± 69.92
VMI 100 keV	17.70 ± 4.17 (p < 0.0001)	65.69 ± 13.23 (p < 0.0001)	138.58 ± 36.45 (p < 0.0001)	38.45 ± 10.35 (p < 0.3330)	242.72 ± 43.23 (p < 0.0001)
VMI 110 keV	17.06 ± 3.87 (p < 0.0001)	68.85 ± 11.41 (p < 0.0001)	122.38 ± 35.48 (p < 0.0001)	37.17 ± 8.93 (p < 0.2916)	228.40 ± 40.42 (p < 0.0001)
VMI 120 keV	17.60 ± 3.69 (p < 0.0001)	74.28 ± 11.00 (p < 0.0001)	112.15 ± 35.58 (p < 0.0001)	38.27 ± 8.25 (p < 0.2629)	224.69 ± 39.46 (p < 0.0001)
VMI 130 keV	18.54 ± 3.60 (p < 0.0001)	79.66 ± 11.21 (p < 0.0001)	112.76 ± 35.44 (p < 0.0001)	40.64 ± 7.90 (p < 0.2652)	233.06 ± 39.04 (p < 0.0001)
VMI 140 keV	19.49 ± 3.53 (p < 0.0001)	85.06 ± 11.57 (p < 0.0001)	117.22 ± 35.33 (p < 0.0001)	42.54 ± 7.92 (p < 0.2746)	244.81 ± 38.62 (p < 0.0001)
VMI 150 keV	20.38 ± 3.50 (p < 0.0001)	90.06 ± 11.98 (p < 0.0001)	121.46 ± 35.36 (p < 0.0001)	44.13 ± 8.03 (p < 0.2749)	255.65 ± 38.41 (p < 0.0001)
VMI 160 keV	21.18 ± 3.47 (p < 0.0001)	93.96 ± 12.33 (p < 0.0001)	124.82 ± 35.52 (p = 0.0001)	45.56 ± 8.35 (p < 0.2751)	264.34 ± 38.49 (p < 0.0001)
VMI 170 keV	21.82 ± 3.46 (p < 0.0001)	97.08 ± 12.69 (p < 0.0001)	128.73 ± 35.56 (p = 0.0002)	46.70 ± 8.39 (p < 0. 2752)	272.50 ± 38.13 (p < 0.0001)
VMI 180 keV	22.40 ± 3.45 (p < 0.0001)	99.67 ± 13.05 (p < 0.0001)	132.07 ± 35.63 (p = 0.0003)	48.19 ± 8.42 (p < 0.2760)	279.92 ± 37.90 (p < 0.0001)
VMI 190 keV	22.86 ± 3.44 (p < 0.0001)	101.83 ± 13.32 (p < 0.0001)	134.73 ± 35.72 (p = 0.0003)	49.37 ± 8.48 (p < 0.2791)	285.94 ± 37.81 (p < 0.0001)
IMAR PI	14.60 ± 4.54 (p < 0.0001)	54.76 ± 14.38 (p < 0.0001)	94.69 ± 35.50 (p < 0.0001)	24.43 ± 5.76 (p < 0.0001)	173.88 ± 37.96 (p < 0.0001)
IMAR 100 keV	8.62 ± 4.16 (p < 0.0001)	33.37 ± 13.09 (p < 0.0001)	51.18 ± 7.01 (p < 0.0001)	18.27 ± 3.72 (p < 0.0001)	102.81 ± 15.91 (p < 0.0001)
IMAR 110 keV	8.37 ± 4.38 (p < 0.0001)	32.67 ± 13.13 (p < 0.0001)	55.33 ± 10.73 (p < 0.0001)	16.97 ± 3.42 (p < 0.0001)	104.97 ± 19.39 (p < 0.0001)
IMAR 120 keV	8.40 ± 4.53 (p < 0.0001)	33.54 ± 13.17 (p < 0.0001)	61.19 ± 14.68 (p < 0.0001)	16.12 ± 3.27 (p < 0.0001)	110.86 ± 22.59 (p < 0.0001)
IMAR 130 keV	8.53 ± 4.68 (p < 0.0001)	34.62 ± 13.20 (p < 0.0001)	60.89 ± 17.60 (p < 0.0001)	15.64 ± 3.15 (p < 0.0001)	111.15 ± 25.33 (p < 0.0001)
IMAR 140 keV	8.7 ± 4.84 (p < 0.0001)	35.66 ± 13.22 (p < 0.0001)	62.90 ± 20.02 (p < 0.0001)	15.35 ± 3.03 (p < 0.0001)	113.91 ± 27.53 (p < 0.0001)
IMAR 150 keV	8.77 ± 4.91 (p < 0.0001)	36.57 ± 13.24 (p < 0.0001)	64.48 ± 21.80 (p < 0.0001)	15.38 ± 2.96 (p < 0.0001)	116.43 ± 29.24 (p < 0.0001)
IMAR 160 keV	8.88 ± 4.99 (p < 0.0001)	37.28 ± 13.25 (p < 0.0001)	65.80 ± 23.33 (p < 0.0001)	15.29 ± 2.90 (p < 0.0001)	118.37 ± 30.65 (p < 0.0001)
IMAR 170 keV	8.99 ± 5.06 (p < 0.0001)	37.88 ± 13.26 (p < 0.0001)	65.58 ± 24.66 (p < 0.0001)	15.25 ± 2.83 (p < 0.0001)	118.71 ± 31.80 (p < 0.0001)
IMAR 180 keV	9.06 ± 5.10 (p < 0.0001)	38.43 ± 13.28 (p < 0.0001)	67.75 ± 25.58 (p < 0.0001)	15.25 ± 2.81 (p < 0.0001)	121.43 ± 32.75 (p < 0.0001)
IMAR 190 keV	9.10 ± 5.14 (p < 0.0001)	38.85 ± 13.29 (p < 0.0001)	67.22 ± 26.32 (p < 0.0001)	15.26 ± 2.81 (p < 0.0001)	121.33 ± 33.53 (p < 0.0001)

Adjusted attenuation closest to 0 shows most favorable artifact reduction. Adjusted noise was calculated for expected lower image noise in high keV and addresses noise without presence of artifacts.

### Qualitative image analysis

**Figure 5:**
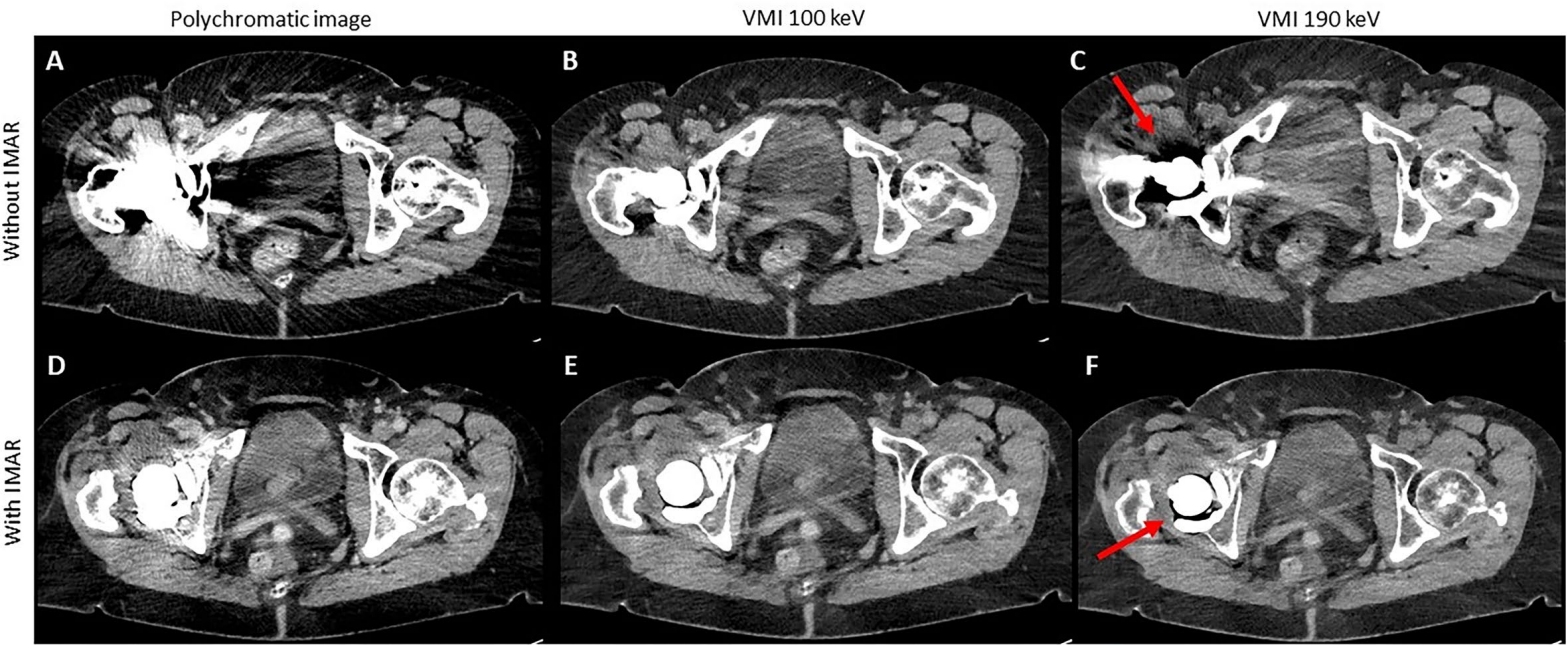
Representative axial polychromatic image (**A**), virtual monoenergetic image (VMI) of 100 keV (**B**), VMI of 190 keV (**C**), polychromatic image with iterative metal artifact reduction (IMAR) (**D**), VMI of 100 keV combined with IMAR (**E**), and VMI of 190 keV in combination with IMAR (**F**) acquired with a photon-counting detector CT (PCD-CT) (window width/window level: 300/40 HU). With increased keV, pronounced hypodense artifacts (arrows) are observed compared to low-keV images.

**Figure 6:**
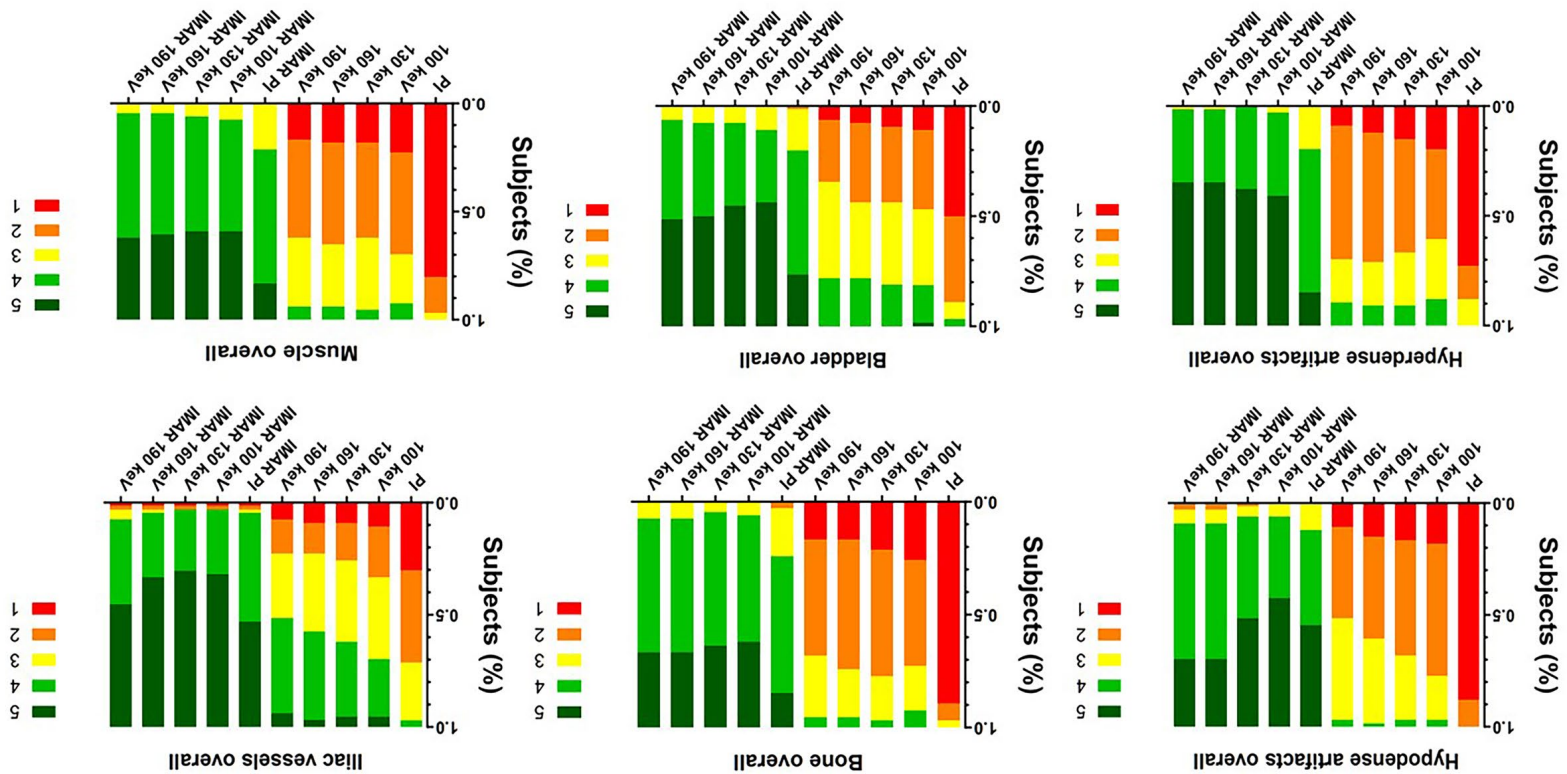
Bar plots show distribution of hypodense artifact extent, hyperdense artifact extent and diagnostic quality rating of bone, bladder, iliac vessels and muscle tissue for polyenergetic reconstruction (PI) and virtual monoenergetic images (VMI) with 100 keV, 130 keV, 160 keV and 190 keV with and without iterative metal artifact reduction (IMAR). Qualitative assessment improved for all investigated image reconstructions compared to polychromatic images (PI). VMI_100keV_ in combination with IMAR achieved best results (e.g. diagnostic quality of the bladder: median PI: 1.5 (range 1–4); VMI_100keV+IMAR_: 5 (3–5); p < 0.0001).

Qualitative rating results are summarized in Supplemental Information 2. Qualitative assessment of muscle (M), bones (BO), bladder (BL) and iliac vessels (IV) significantly improved compared to conventional images (median PI: M 1 (1–3), BO 1 (1–3), BL 1.5 (1–4); IV 2 (1–4); median VMI_100keV+IMAR_: M 4 (3–5), BO 4 (3–5), BL 5 (3–5); IV 5 (2–5); p < 0.05; Supplemental Information 2). Hyperdense artifacts were subjectively reduced, scoring a 5 (3–5, p < 0.05) for all reconstructions combining VMI and IMAR. For the PI the score was 1 (1–3) and VMI_190keV_ scored 2 (1–4; p < 0.05). For hypodense artifacts, PI scored 1 (1–2), VMI_190keV_ scored 2 (1–4; p < 0.05) and combination of VMI_100keV_ and IMAR scored 5 (3–5; p < 0.05). In many cases especially hypodense artifacts adjacent to the prosthesis increased with higher keV VMI (Fig. 5). Rating results for all criteria by each rater are shown in Fig. 6. Overall, raters favored VMI_100keV+IMAR_ in 62.12% of all scans (41/66) and VMI_130keV+IMAR_ in 28.79% of the examinations (19/66).

Interrater agreement was excellent with an overall ICC value of 0.944 [95% confidence interval (CI) 0.939–0.949]. Respective ICC was 0.963 (95% CI 0.955–0.970) for the extent of hypodense artifacts, 0.956 (95% CI 0.946–0.965) for the extent of hyperdense artifacts, 0.953 (95% CI 0.942–0.962) for assessment of muscle tissue, 0.976 (95% CI 0.970–0.981) for bone, 0.915 (95% CI 0.895–0.931) for assessment of the bladder and 0.909 (95% CI 0.889–0.926) for assessment of the iliac vessels.

## Discussion

This study evaluates PCD-CT artifact reduction capabilities regarding hip implants in clinical imaging. The main findings are that VMI_100keV_ in combination with IMAR deliver best results regarding artifact-reduction and diagnostic quality. Every combination of VMI and IMAR led to artifact reduction compared to conventional polychromatic images, with low keV VMI images demonstrating best contrast, especially for vascular delineation in scans with iodine contrast.

As hip arthroplasty and associated revision surgery procedures are projected to increase, there is a growing need for precise, fast and artifact-free imaging including dedicated scans for evaluation of hip arthroplasty-associated pathologies^16^. PCD-CT is an emerging technology with numerous potential advantages compared with EID-CT based on the direct detection and weighting of every photon^17,18^. Employing energy-thresholds allows for elimination of image background noise; virtual higher-energy image reconstructions are less affected by artifacts typically originating from lower energy photons^19^. High photon sensitivity is a further inert advantage of PCD-CT; diagnostic image quality is achievable with lower tube current, than necessary with EID CT.

Several studies have investigated the effects of VMI and IMAR for reduction of artifacts in dual energy EID-CT^9,20–26^. A recent study investigating split-filter EID-CT with images reconstructed at 120 kVp-equivalent, reported that PI images in combination with IMAR leads to best artifact reduction^15^. In contrast, most previous EID-CT studies reported best artifact reduction for high-energy VMI in combination with IMAR^23–26^.

**Figure 7:**
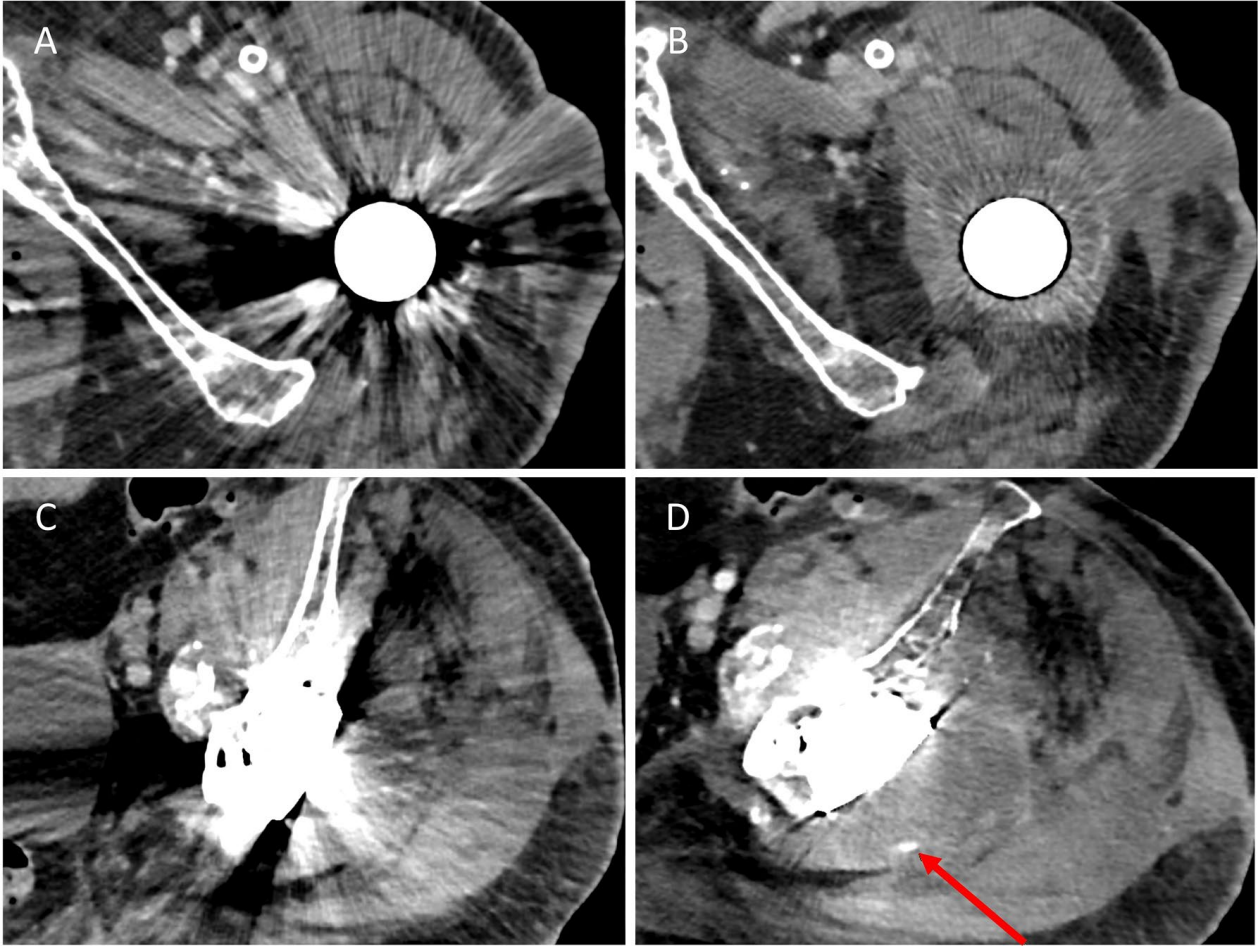
Representative axial images of a periprosthetic fluid collection in a polychromatic reconstruction (**A**) and virtual monoenergetic image of 100 keV in combination with iterative metal artifact reduction (IMAR) (**B**), as well as polychromatic images without (**C**) and with IMAR (**D**) of a hematoma with blood pooling in venous phase (arrow). Both findings would have been concealed without artifact reduction techniques.

Even though this study did not compare PCD-CT to EID-CT, images achieved were of high diagnostic quality and therefore offer a feasible solution for patients with hip prosthesis suspected of pathologies masked by implant-associated artifacts (Fig. 7).

As signal acquisition of PCD-CT differs from dual energy EID-CT, with PCD-CT technology enabling imaging with lower noise and higher spatial resolution, lower energy PCD-CT VMI images are expected to have less noise and higher image quality. In fact, in PCD-CT artifact extent remains relatively stable between 120 and 190 keV, with 190 keV images showing severely reduced contrast; Images reconstructed at 100 keV combined with IMAR resulted in best artifact reduction and showed superior contrast compared to high-keV images.

A recent phantom study on PCD-CT reported best artifact reduction for 100 keV at scans performed with 120 kVp, which is in line with the results of this study^27^. As recently reported for EID-CT, in PCD-CT IMAR is superior to VMI for the reduction of extensive artifacts but may lead to distortion and blurring. VMI effectively reduces minor artifacts without distorting images^15,23^.

Laukamp et al. reported optimal keV reconstructions for artifact reduction in EID-CT depending on the affected tissue; 200 keV for bone, 140 keV for soft tissue^9^. This could not be confirmed for PCD-CT in the current study. Quantitatively, there were minor, insignificant deviations of adjusted attenuation for muscle and bladder towards VMI with higher keV. Nevertheless, VMI_100 keV_ in combination with IMAR led to optimal results for both quantitative and qualitative assessment of artifact reduction and image quality.

As reported previously^15,23,28^, combining IMAR and high keV VMI led to the appearance of new hypodense artifacts adjacent to implants, suggesting a local overcorrection of artifacts. These new artifacts intensify with higher keV VMI and thereby limit diagnostic assessment of surrounding tissue.

There are limitations to the study. Study design was monocentric and therefore only a small cohort of patients was retrospectively included. No distinction was made between unilateral and bilateral hip prostheses. Furthermore, prosthesis composition was not taken into account. As stated before, based on the material and shape varying artifact extent can be assumed^3,29^. Further multi-center evaluation on larger cohorts, especially focusing on patients needing a hip prosthesis revision should be part of further subsequent studies.

Overall, the current results show that the combination of IMAR and VMI are the most potent tool for reduction of even extensive hip prosthesis associated artifacts in PCD-CT. The combination of IMAR and VMI improves diagnostic readability and thus may enable the detection of pathologies that would otherwise be concealed by artifacts in conventional CT images.

## Supplementary Information

Supplementary Information 1.

Supplementary Information 2.

## Data Availability

The anonymized datasets generated during and analyzed during the current study are available from the corresponding author on reasonable request. Due to local privacy laws, CT images can not be provided as theoretically there is a risk of identification of personal information in pseudo-anonymized CT datasets.
