# Supplementary material for: Combining iterative metal artifact reduction and virtual monoenergetic images severely reduces hip prosthesis-associated artifacts in photon-counting detector CT
Source: Sci Rep. 2023 Jun 2;13:8955. doi: 10.1038/s41598-023-35989-6 (PMC10238372; doi:10.1038/s41598-023-35989-6)
Supplement: Supplementary file 1 — Supplementary Information 1. [file 41598_2023_35989_MOESM1_ESM.docx]

|  | Hypodense artifacts [HU] | Hyperdense artifacts [HU] | Muscle tissue (artifacts) [HU] | Muscle tissue (without artifacts) [HU] | Bone tissue (artifacts) [HU] | Bone tissue (without artifacts) [HU] | Bladder (artifacts) [HU] | Bladder (without artifacts) [HU] |
| --- | --- | --- | --- | --- | --- | --- | --- | --- |
| PI | -424.97 ± 79.16 | 661.58 ± 159.78 | 74.03 ± 69.19 | 42.62 ± 21.04 | 82.18 ± 124.34 | 88.11 ± 50.21 | -82.43 ± 52.51 | 34.96 ± 23.01 |
| VMI 100 keV | -203.52 ± 72.8  (p = 0.0003) | 282.55 ± 95.28  (p < 0.0001) | 79.76 ± 33.68  (p > 0.9999) | 43.47 ± 15.98  (p = 0.5391) | -8.00 ± 71.06  (p = 0.0600) | 54.26 ± 31.59  (p < 0.0001) | -4.04 ± 31.48  (p < 0.0001) | 28.01 ± 16.37  (p = 0.3330) |
| VMI 110 keV | -168.52 ± 72.7  (p = 0.0003) | 219.62 ± 90.91  (p < 0.0001) | 80.46 ± 32.82  (p > 0.9999) | 43.65 ± 15.76  (p = 0.4990) | -22.09 ± 68.85  (p = 0.0708) | 48.91 ± 30.14  (p < 0.0001) | 7.80 ± 30.65  (p < 0.0001) | 26.80 ± 16.20  (p = 0.2916) |
| VMI 120 keV | -142.81 ± 72.95  (p = 0.0003) | 178.64 ± 90.38  (p < 0.0001) | 80.95 ± 33.24  (p > 0.9999) | 43.79 ± 15.64  (p = 0.4776) | -31.72 ± 68.50  (p = 0.0626) | 45.10 ± 29.14  (p < 0.0001) | 16.50 ± 30.41  (p < 0.0001) | 25.92 ± 16.18  (p = 0.2629) |
| VMI 130 keV | -123.70 ± 73.38  (p = 0.0003) | 145.97 ± 91.05  (p < 0.0001) | 81.32 ± 34.08  (p > 0.9999) | 43.86 ± 15.54  (p = 0.4759) | -38.83 ± 68.83  (p = 0.0652) | 42.21 ± 28.45  (p < 0.0001) | 22.94 ± 30.48  (p < 0.0001) | 25.29 ± 16.22  (p = 0.2652) |
| VMI 140 keV | -109.16 ± 73.83  (p = 0.0003) | 120.99 ± 92.2  (p < 0.0001) | 81.62 ± 34.97  (p > 0.9999) | 43.95 ± 15.48  (p = 0.4618) | -44.21 ± 69.49  (p = 0.0626) | 40.02 ± 27.95  (p < 0.0001) | 27.89 ± 30.79  (p < 0.0001) | 24.77 ± 16.28  (p = 0.2746) |
| VMI 150 keV | -97.94 ± 74.28  (p = 0.0003) | 101.74 ± 93.31  (p < 0.0001) | 81.87 ± 35.83  (p > 0.9999) | 44.01 ± 15.45  (p = 0.4525) | -48.41 ± 70.22  (p = 0.0601) | 38.30 ± 27.58  (p < 0.0001) | 31.67 ± 31.2  (p < 0.0001) | 24.41 ± 16.34  (p = 0.2652) |
| VMI 160 keV | -89.03 ± 74.77  (p = 0.0003) | 86.58 ± 94.35  (p < 0.0001) | 82.05 ± 36.57  (p > 0.9999) | 44.04 ± 15.39  (p = 0.4553) | -51.77 ± 70.9  (p = 0.0589) | 36.99 ± 27.31  (p < 0.0001) | 34.47 ± 31.54  (p < 0.0001) | 24.10 ± 16.40  (p = 0.2650) |
| VMI 170 keV | -82.1 ± 75.06  (p = 0.0003) | 74.50 ± 95.32  (p < 0.0001) | 82.15 ± 37.19  (p > 0.9999) | 44.09 ± 15.38  (p = 0.4462) | -54.35 ± 71.52  (p = 0.0575) | 35.92 ± 27.08  (p < 0.0001) | 37.10 ± 31.97  (p < 0.0001) | 23.86 ± 16.46  (p = 0.2623) |
| VMI 180 keV | -76.28 ± 75.34  (p = 0.0003) | 64.69 ± 96.11  (p < 0.0001) | 82.32 ± 37.75  (p > 0.9999) | 44.11 ± 15.34  (p = 0.4451) | -56.55 ± 72.05  (p = 0.0553) | 35.08 ± 26.93  (p < 0.0001) | 39.02 ± 32.29  (p < 0.0001) | 23.65 ± 16.50  (p = 0.2561) |
| VMI 190 keV | -71.64 ± 75.53  (p = 0.0002) | 56.79 ± 96.82  (p < 0.0001) | 82.4 ± 38.2  (p > 0.9999) | 44.14 ± 15.34  (p = 0.4445) | -58.23 ± 72.55  (p = 0.0541) | 34.39 ± 26.78  (p < 0.0001) | 40.57 ± 32.56  (p < 0.0001) | 23.49 ± 16.54  (p = 0.2560) |
| VMI IMAR PI | 2.21 ± 34.12  (p < 0.0001) | 171.46 ± 109.36  (p < 0.0001) | 58.98 ± 33.48  (p = 0.7376) | 44.41 ± 18.88  (p = 0.1565) | 123.08 ± 75.57  (p = 0.9578) | 86.53 ± 47.31  (p = 0.6674) | 15.79 ± 19.80  (p < 0.0001) | 33.93 ± 21.36  (p = 0.7579) |
| IMAR + VMI 100 keV | 10.69 ± 27.12  (p < 0.0001) | 65.95 ± 59.39  (p < 0.0001) | 56.63 ± 23.32  (p = 0.6476) | 41.88 ± 14.70  (p = 0.6188) | 53.86 ± 42.81  (p = 0.6835) | 54.29 ± 32.67  (p < 0.0001) | 15.73 ± 12.92  (p < 0.0001) | 23.27 ± 14.86  (p = 0.3067) |
| IMAR + VMI 110 keV | 12.84 ± 26.82  (p < 0.0001) | 49.63 ± 59.87  (p < 0.0001) | 56.41 ± 22.89  (p = 0.6388) | 41.48 ± 14.52  (p = 0.3870) | 42.75 ± 44.08  (p = 0.6592) | 48.98 ± 31.07  (p < 0.0001) | 15.00 ± 12.72  (p < 0.0001) | 21.49 ± 14.63  (p = 0.2297) |
| IMAR + VMI 120 keV | 14.40 ± 26.64  (p < 0.0001) | 38.12 ± 61.53  (p < 0.0001) | 56.26 ± 22.81  (p = 0.6213) | 41.2 ± 14.42  (p = 0.3357) | 34.67 ± 45.38  (p = 0.6213) | 45.10 ± 29.96  (p < 0.0001) | 14.49 ± 12.58  (p < 0.0001) | 20.21 ± 14.49  (p = 0.2291) |
| IMAR + VMI 130 keV | 15.58 ± 26.55  (p < 0.0001) | 29.54 ± 63.34  (p < 0.0001) | 56.23 ± 22.86  (p = 0.5873) | 40.99 ± 14.33  (p = 0.4688) | 28.57 ± 46.51  (p = 0.5483) | 42.25 ± 29.18  (p < 0.0001) | 14.31 ± 12.53  (p < 0.0001) | 19.23 ± 14.42  (p = 0.2072) |
| IMAR + VMI 140 keV | 16.50 ± 26.47  (p < 0.0001) | 23.01 ± 64.92  (p < 0.0001) | 56.14 ± 22.96  (p = 0.5774) | 40.82 ± 14.26  (p = 0.3012) | 23.98 ± 47.42  (p = 0.5369) | 40.06 ± 28.62  (p < 0.0001) | 13.74 ± 12.47  (p < 0.0001) | 18.50 ± 14.41  (p = 0.2052) |
| IMAR + VMI 150 keV | 17.18 ± 26.44  (p < 0.0001) | 17.97 ± 66.26  (p < 0.0001) | 56.06 ±22.99  (p = 0.5751) | 40.71 ± 14.23  (p = 0.2800) | 20.43 ± 48.16  (p = 0.5250) | 38.35 ± 28.18  (p < 0.0001) | 13.49 ± 12.44  (p < 0.0001) | 17.93 ± 14.37  (p = 0.1976) |
| IMAR + VMI 160 keV | 17.75 ± 26.40  (p < 0.0001) | 13.96 ± 67.47  (p < 0.0001) | 56.02 ± 23.08  (p = 0.5722) | 40.59 ± 14.2  (p = 0.2598) | 17.62 ± 48.74  (p = 0.5102) | 37.06 ± 27.85  (p < 0.0001) | 13.36 ± 12.44  (p < 0.0001) | 17.45 ± 14.37  (p = 0.1932) |
| IMAR + VMI 170 keV | 18.17 ± 26.38  (p < 0.0001) | 10.80 ± 68.43  (p < 0.0001) | 55.97 ± 23.15  (p = 0.5718) | 40.51 ± 14.16  (p = 0.2475) | 15.36 ± 49.23  (p = 0.5021) | 37.32 ± 27.27  (p < 0.0001) | 13.18 ± 12.42  (p < 0.0001) | 17.13 ± 14.37  (p = 0.1828) |
| IMAR + VMI 180 keV | 18.54 ± 26.36  (p < 0.0001) | 8.3 ± 69.20  (p < 0.0001) | 55.99 ±23.21  (p = 0.5713) | 40.46 ± 14.14  (p = 0.2400) | 13.57 ± 49.65  (p = 0.4888) | 35.28 ± 27.41  (p < 0.0001) | 13.07 ± 12.41  (p < 0.0001) | 16.82 ± 14.36  (p = 0.1757) |
| IMAR + VMI 190 keV | 18.83 ± 26.35  (p < 0.0001) | 6.23 ± 69.91  (p < 0.0001) | 55.95 ± 23.23  (p = 0.5707) | 40.4 ± 14.13  (p = 0.2312) | 12.17 ± 50.01  (p = 0.4798) | 35.89 ± 26.92  (p < 0.0001) | 12.99 ± 12.41  (p < 0.0001) | 16.60 ± 14.35  (p = 0.1638) |

**Supplementary Information 1:** Mean attenuation values and standard deviation within defined regions of interest tissue for polyenergetic reconstruction (PI) and virtual monoenergetic images (VMI) with and without iterative metal artifact reduction (IMAR).
