# Supplementary material for: Combining iterative metal artifact reduction and virtual monoenergetic images severely reduces hip prosthesis-associated artifacts in photon-counting detector CT
Source: Sci Rep. 2023 Jun 2;13:8955. doi: 10.1038/s41598-023-35989-6 (PMC10238372; doi:10.1038/s41598-023-35989-6)
Supplement: Supplementary file 2 — Supplementary Information 2. [file 41598_2023_35989_MOESM2_ESM.docx]

|  | PI (IQR) | VMI 100 keV (IQR) | VMI 130 keV  (IQR) | VMI 160 keV  (IQR) | VMI 190 keV  (IQR) | IMAR PI (IQR) | IMAR + VMI 100 keV (IQR) | IMAR + VMI 130 keV (IQR) | IMAR + VMI 160 keV (IQR) | IMAR + VMI 190 keV (IQR) | ICC (95% CI) |
| --- | --- | --- | --- | --- | --- | --- | --- | --- | --- | --- | --- |
| Extent of hypodense artifacts | 1 (1-1) | **2** (2-2)  (p < 0.0001) | **2** (2-3)  (p < 0.0001) | **2** (2-3)  (p < 0.0001) | **2** (2-3)  (p < 0.0001) | **4** (4-5)  (p < 0.0001) | **5** (4-5)  (p < 0.0001) | **4** (4-5)  (p < 0.0001) | **4** (4-5)  (p < 0.0001) | **4** (4-5)  (p < 0.0001) | 0.963 (0.955-0.970) |
| Extent of hyperdense artifacts | 1 (1-2) | **2** (2-3)  (p < 0.0001) | **2** (2-3)  (p < 0.0001) | **2** (2-3)  (p < 0.0001) | **2** (2-3)  (p < 0.0001) | **4** (4-4)  (p < 0.0001) | **5** (4-5)  (p < 0.0001) | **5** (4-5)  (p < 0.0001) | **5** (4-5)  (p < 0.0001) | **5** (4-5)  (p < 0.0001) | 0.956 (0.946-0.965) |
| Diagnostic quality of muscle tissue | 1 (1-1) | **2** (2-3)  (p < 0.0001) | **2** (2-3)  (p < 0.0001) | **2** (2-3)  (p < 0.0001) | **2** (2-3)  (p < 0.0001) | **4** (4-4)  (p < 0.0001) | **4** (4-5)  (p < 0.0001) | **4** (4-5)  (p < 0.0001) | **4** (4-5)  (p < 0.0001) | **4** (4-5)  (p < 0.0001) | 0.953 (0.942-0.962) |
| Diagnostic quality of bone tissue | 1 (1-1) | **2** (1-3)  (p < 0.0001) | **2** (2-2)  (p < 0.0001) | **2** (2-3)  (p < 0.0001) | **2** (2-3)  (p < 0.0001) | **4** (3.75-4)  (p < 0.0001) | **4** (4-5)  (p < 0.0001) | **4** (4-5)  (p < 0.0001) | **4** (4-5)  (p < 0.0001) | **4** (4-5)  (p < 0.0001) | 0.976 (0.970-0.981) |
| Diagnostic quality of the bladder | 1.5 (1-2) | **3** (2-3)  (p < 0.0001) | **3** (2-3)  (p < 0.0001) | **3** (2-3)  (p < 0.0001) | **3** (2-3)  (p < 0.0001) | **4** (4-4)  (p < 0.0001) | **5** (4-5)  (p < 0.0001) | **5** (4-5)  (p < 0.0001) | **4.5** (4-5)  (p < 0.0001) | **4** (4-5)  (p < 0.0001) | 0.915 (0.895-0.931) |
| Diagnostic quality of the iliac vessels | 2 (1-3) | **3** (2-4)  (p < 0.0001) | **3** (2-4)  (p < 0.0001) | **3** (3-4)  (p < 0.0001) | **3** (3-4)  (p < 0.0001) | **4** (4-5)  (p < 0.0001) | **5** (4-5)  (p < 0.0001) | **5** (4-5)  (p < 0.0001) | **5** (4-5)  (p < 0.0001) | **5** (4-5)  (p < 0.0001) | 0.909 (0.889-0.926) |

**Supplementary Information 2**: Median and interquartile range (IQR) of overall qualitative image ratings for polyenergetic reconstruction (PI) and virtual monoenergetic images (VMI) with and without iterative metal artifact reduction (IMAR). Intraclass correlation coefficient (ICC) estimates and their 95% confident intervals were calculated. ICC calculation is based on a mean-rating (k=2), consistency, two-way mixed-effects model.
